# Early stent thrombosis: a complication that we thought had disappeared

**DOI:** 10.1093/ehjcr/ytae184

**Published:** 2024-04-09

**Authors:** Amine Ech-chenbouli, Badre Elboussaadani, Malak Benabdellah, Zainab Raissuni

**Affiliations:** Cardiology Department of Mohamed 6th University Hospital Tangier Morocco, Tangier’s University Hospital Cathlab, Abdelmalek Saadi University Faculty of Medicine and Pharmacy of Tangier, BP 1818 KM 17 route de rabat, Tangier, Morocco; Cardiology Department of Mohamed 6th University Hospital Tangier Morocco, Tangier’s University Hospital Cathlab, Abdelmalek Saadi University Faculty of Medicine and Pharmacy of Tangier, BP 1818 KM 17 route de rabat, Tangier, Morocco; Cardiology Department of Mohamed 6th University Hospital Tangier Morocco, Tangier’s University Hospital Cathlab, Abdelmalek Saadi University Faculty of Medicine and Pharmacy of Tangier, BP 1818 KM 17 route de rabat, Tangier, Morocco; Cardiology Department of Mohamed 6th University Hospital Tangier Morocco, Tangier’s University Hospital Cathlab, Abdelmalek Saadi University Faculty of Medicine and Pharmacy of Tangier, BP 1818 KM 17 route de rabat, Tangier, Morocco

## Case presentation

A 45-year-old women without any cardiovascular risk factors was referred to our Cath lab for probable stent thrombosis 5 days after initial angioplasty for an inferior ST elevation myocardial infarction (STEMI) with a 2.5 × 18 mm Rapamycin Eluting stent with biodegradable polymer in mid right coronary artery. The patient was then discharged with apparently good angiographic result and was prescribed clopidogrel + aspirin double antiplatelet therapy (DAPT) (the patient could not afford a potent P2Y12 (ticagrelor or prasugrel) due to its cost and lack of medical insurance), high dose of atorvastatin (80 mg o.d) and 5 mg of ramipril o.d.

**Figure 1:**
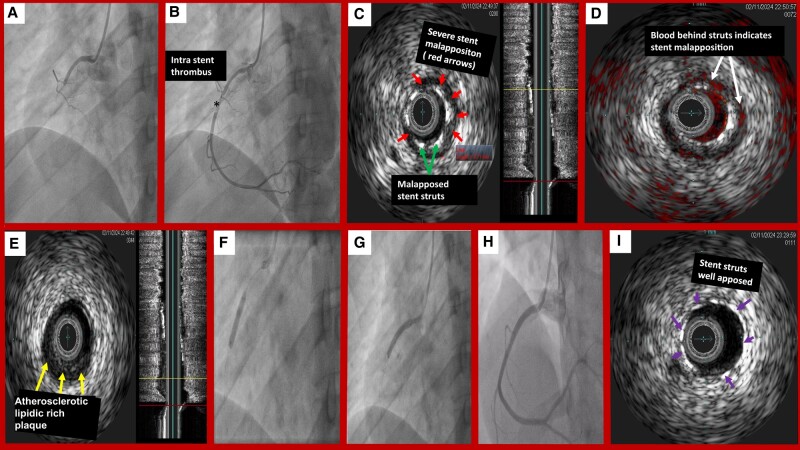
Right coronary angiography showing total acute occlusion of the mid coronary artery at the stent implantation site (stent thrombosis): white asterisk *(Panel A)* with a large intra-stent thrombus: black asterisk *(Panel B).* Intravascular ultrasound (IVUS) showing severe stent malapposition: red arrows point to locations of malapposition with a maximal malapposition length of 7 mm and clearly an undersized stent (green arrows point to stent struts) *(Panel C).* Chroma flow IVUS showing blood behind struts (white arrows) *(Panel D)*. IVUS showing Proximal lipidic rich plaque missed in the first angioplasty: yellow arrows *(Panel E).* Right coronary angiography showing overexpansion of the thrombosed stent and addition of a proximal stent *(Panel F and G)*. Achievement of a good angiographic result (*Panel H)* with good apposition of the stent struts (purple arrows point to well apposed struts) *(Panel I).*

Coronary Angiography confirmed stent thrombosis with large intra-stent thrombus (*Figure 1*, *Panel A* and *B*), Intravascular ultrasound (IVUS) showed severe stent malapposition with an undersized stent and geographic missing of a proximal lipidic rich plaque (*Figure 1*, *Panel C*, *D*, and *E*).

Treatment consisted of overexpansion of the stent with a 4.0 × 12 mm non-compliant balloon (NCB) and addition of a 3.5 × 18 mm drug eluting stent (DES) proximal to the previously implanted one (*Figure 1*, *Panel F* and *G*) to achieve good angiographic result (*Figure 1*, *Panel H*) and good apposition of the stent on IVUS (*Figure 1*, *Panel I*). Patient was discharged 2 days after and was prescribed ticagrelor + aspirin.

## Supplementary Material

ytae184_Supplementary_Data

## Data Availability

Data underlying this article are available in the article and its online supplementary

## Supplementary material

**Consent**: The authors confirm that written consent for submission and publication of this Image and associated text has been received from the patient in line with the Committee on Publication Ethics (COPE) guidelines.

**Funding**: None declared.

